# A native mass spectrometry approach to qualitatively elucidate interfacial epitopes of transient protein–protein interactions†

**DOI:** 10.1039/d4cc01251h

**Published:** 2024-05-13

**Authors:** Clinton G. L. Veale, Abir Chakraborty, Richwell Mhlanga, Fernando Albericio, Beatriz G. de la Torre, Adrienne L. Edkins, David J. Clarke

**Affiliations:** a Department of Chemistry, University of Cape Town Rondebosch Cape Town 7701 South Africa clinton.veale@uct.ac.za; b The Biomedical Biotechnology Research Unit (BioBRU), Department of Biochemistry and Microbiology, Rhodes University Makhanda South Africa; c School of Chemistry and Physics, University of KwaZulu-Natal Westville South Africa; d School of Laboratory Medicine and Medical Sciences, College of Health Sciences, University of KwaZulu-Natal South Africa; e EaStCHEM School of Chemistry, University of Edinburgh Joseph Black Building, David Brewster Road Edinburgh EH93FJ UK dave.clarke@ed.ac.uk

## Abstract

Native mass spectrometric analysis of TPR2A and GrpE with unpurified peptides derived from limited proteolysis of their respective PPI partners (HSP90 C-terminus and DnaK) facilitated efficient, qualitative identification of interfacial epitopes involved in transient PPI formation. Application of this approach can assist in elucidating interfaces of currently uncharacterised transient PPIs.

## Abstract 2

Here we demonstrate a new approach in which native mass spectrometry and limited proteolysis is used in concert to rapidly identify interfacial peptides responsible for mediating a transient Protein–Protein Interaction.

## Graphical abstract

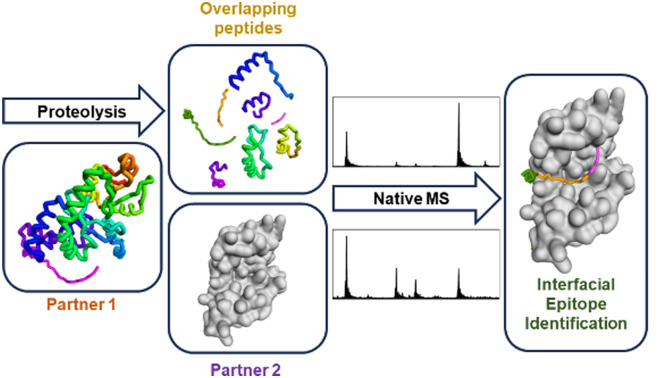

The central mediatory role of protein–protein interactions (PPIs) in biological processes offers significant opportunity to unravel details of disease progression and expand druggable chemical space.^1^ Many biological functions including, signalling networks and regulation of biochemical pathways, rely on a subset of PPIs, known as transient PPIs, where interfacial associations are comparatively weak and shorter-lived.^2^ A full understanding of the biological function and mechanism of interactions within transient PPIs as well as the development of PPI modulators requires detailed structural characterisation of binding interfaces. This includes locating and identifying interacting domains, amino acid sequences and so-called hot-spot regions.^3^ Compared to strong/permeant PPIs, such as antibody-antigen interactions, transient PPIs are dynamic associations, whose specific, but low affinity associations are commonly mediated by the interaction between a domain and a disordered short linear motifs (SLiMs).^4^ While PPI transience is a key for property required for mediating signalling networks,^5^ their instability makes studying transient PPIs a considerable challenge.

Fortuitously the dominant peptide-protein interactions (Pep-PIs) to be studied in isolation as reduced complexity PPI model systems for interfacial characterisation and inhibitor design.^6^

Native mass spectrometry (MS), in which native state solution-phase structural information is transmitted into the gas phase, facilitates the elucidation and quantification of weak biomolecular interactions.^7,8^ Its inherent speed, sensitivity, low sample consumption and comparably simpler experimental set-up in comparison to X-ray crystallography, NMR or cryo-EM has seen its increasing up-take in structural biology.^9^ Importantly, the ability of native MS to confidently discern between binding species based on specific alterations of the mass-to-charge ratio (*m*/*z*) is particularly useful for chemical biology and drug discovery applications and has seen it utilised to directly observe Pep-PIs as gas-phase PPI proxies capable of identifying PPI modulators.^10–12^ Nevertheless, the *in vitro* identification of natural interfacial peptides required to construct a suitably representative Pep-PI is another substantial challenge, typically requiring some format of peptide scanning and, ultimately, the synthesis of a library of overlapping peptides.^13,14^ Alternatively, biologically inspired methodologies such as phage display are uniquely powerful means of identifying binding epitopes. However, its application to PPI interfaces requires the construction of custom phage display libraries, which suitably represent the partner protein in question.^15^ Therefore, the simplification of epitope mapping methodologies for PPI interfacial elucidation becomes a function of efficiently generating predictable peptides representative of a partner protein combined with the ability to detect and compare their relative interaction with the corresponding partner protein.

In a study investigating the strong antibody–antigen interaction of Amyloid β-Protein (Aβ) and an anti-Aβ antibody, Lu *et al.* utilised limited digestion proteolysis to excise an 11 amino acid linear epitope from the 40 amino acid Aβ 1–40 peptide, which through native MS was confirmed to bind to the Fab region of the anti-Aβ antibody.^16^

We reasoned that a similar approach might be suitable for characterising transiently interacting proteins, where limited proteolysis would generate a suite of overlapping peptides representative of one partner protein analyte. We further surmised that a reasonably selective and predictable proteolytic enzyme would allow a crude proteolytic mixture to be directly incubated with the corresponding partner protein and analysed under native MS conditions. Here, we envisioned binding peptides could be identified directly from the crude peptide mixtures from the Δ*m*/*z* emanating from their predictable mass signature. Identification of both binding and non-binding peptides would provide an efficient and rapid means of identifying binding regions and estimating the likely minimal linear epitope responsible for the greatest contribution toward transient PPI interaction formation and further streamlining processes for hot-spot identification. First, we applied this approach to the transient PPI between HSP90 and its co-chaperone HOP. The HOP-HSP90 PPI facilitates the folding of client proteins, which include a host of signalling intermediates and transcription factors, many of which are associated with invasive or aggressive tumours.^17^ The HOP-HSP90 interaction is known to be primarily mediated *via* interaction of the TPR2A domain of HOP with a MEEVD containing SLiM found on the HSP90-C terminus.^18^

Analysis of apo TPR2A under native MS conditions resulted in a charge state distribution consistent with a 20.6 kDa monomer (Fig. S1A, ESI†). Limited proteolysis of the HSP90-C terminal domain (HSP90-C) was conducted using agarose-supported trypsin in a 100 mM NH_4_OAc solution, from which 2 μL aliquots were removed following 1 h, 2 h and 18 h of digestion. Aliquots were directly incubated with TPR2A without further modification and analysed under native MS conditions using high-resolution nESI FT-ICR MS (Fig. 1). At the 1 h timepoint, four distinct peptide binding species (1–4, Table S1, ESI†) were observed, with peptides 1, 2 and 4 appearing as relatively minor TPR2A-binding species and peptide 3 in the largest relative abundance across all charge states (Fig. 1 and Table S2, ESI†). Binding species were unambiguously identified through Δ*m*/*z* analysis compared to the apo peak combined with high-resolution MS analysis of the isotope distributions of protein-peptide peaks (Fig. S2 and Table S1, ESI†). In addition, collision-induced MSMS could be used to dissociate the gas phase protein-peptide complex and directly measure the mass of the free peptide. From these data, 1 was identified as the known interfacial MEEVD peptide and 3 and 4 as larger peptides containing the MEEVD motif. Interestingly, peptide 2 was a non-MEEVD-containing analogue of 3. Direct evidence of the binding of this region is particularly significant since a recent study utilising cryo-EM to resolve a GR–HSP90–HSP70–HOP complex omitted the majority of the sequence of peptide 2 (Fig. S3A and S4, ESI†) from its structure,^19^ possibly due to its intrinsic disorder. The 2 h timepoint saw an incremental increase in the abundance of the smaller peptide 1 and 2, while peptides derived after 18 h of digestion saw more substantial changes, where the complexes of peptides 1 and 2 with TPR2A were substantially more abundant (Fig. 1). The TPR2A–3 complex had reduced in abundance to be roughly equal to the TPR2A–1 complex (Fig. 1 and Table S2, ESI†). The TPR2A – peptide 4 complex was absent after 18 h. This was confirmed by an LC-MS analysis of the HSP90-C peptide mixture following 18 h of tryptic digestion, which did not contain peptide 4. However, peptides 1–3 were all identifiable, alongside five additional non-binding peptides, which together covered a large proportion of the HSP90-C sequence (Fig. S3A and Table S1, ESI†). Thus, the peptide populations at this timepoint are entirely consistent with our native MS observations. Our data also suggested that varying proteolytic digestion time had little impact on the qualitative identification of binding peptides. However, increased digestion times, and resultant generation of smaller peptides, at the expense of longer binding peptides, provided higher resolution information of potential hot spots on the interfacial SLiM. We progressed to assessing the impact that introducing interface competing ligands (Fig. S5, ESI†) might have on the binding fingerprint of TPR2A with the 18 h tryptic digest of HSP90-C (Fig. 2). Incubation with Ac-MEEVD (5) unsurprisingly resulted in competition for binding with both MEEVD-containing peptide 1 and 3 complexes, respectively, leading to a reduction in their relative abundances (Fig. 2 and Table S2, ESI†). However, it was noticeable that peptides 2 and 5 could bind simultaneously, and the abundance of the TPR2A–2 complex was virtually unaffected by the presence of peptide 5. A tetrazole-containing Ac-MEEVD analogue (Ac-METrVD, 6), which we had previously disclosed as a HOP-HSP90 PPI inhibitor,^10,20^ had a far more pronounced effect on the abundance of peptide 1 and 3 complexes, and while a minor ternary complex with TPR2A and peptides 2 and 6 was observed, peptide 6 was also able to substantially disrupt the TPR2A–2 complex (Fig. 2). Significantly, this indicates that 6 can disrupt the PPI interface beyond the MEEVD interaction site, which likely underpins its previously reported PPI inhibitory activity, in contrast to 5, which shows little PPI inhibitory activity.^10^ This further suggests that the secondary interfacial region is important for discerning between interfacial mimics and potential PPI inhibitors.

**Fig. 1:**
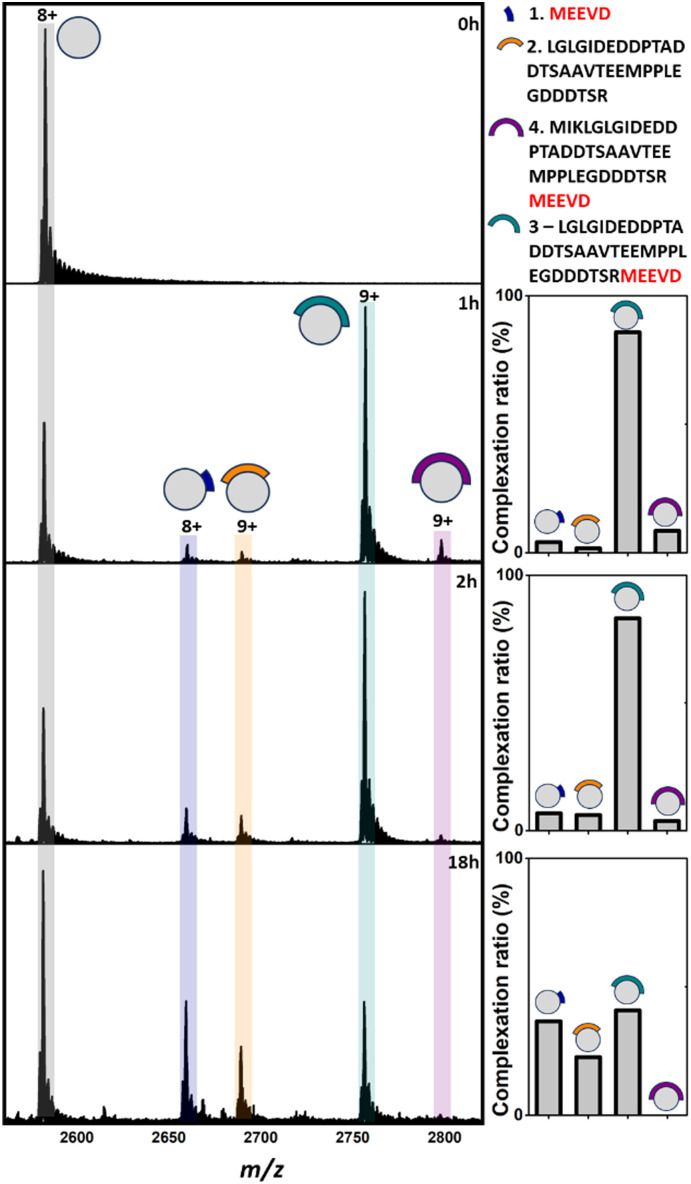
Native mass spectra of apo TPR2A (0 h) and TPR2A incubated with HSP90-C terminal peptides derived from 1 h, 2 h and 18 h of tryptic digestion. In addition to apo TPR2A (*m*/*z* 2580.2, [M + 8H]^8+^), TPR2A complexes can be observed for three MEEVD containing peptides (*m*/*z* 2657.8, [Mb7·1 + 8H]^8+^; *m*/*z* 2754.7, [M·3 + 9H]^9+^; *m*/*z* 2796.0 [M·4 + 9H]^9+^) and one without MEEVD (*m*/*z* 2687.7 [M·2 + 9H]^9+^). Bar graphs alongside spectra highlight the alteration of complexation ratios between TPR2A and peptides 1–4 over the experimental time course. The peptide sequence key is shown on the top right. Full spectra can be found in Fig. S1 (ESI†).

**Fig. 2:**
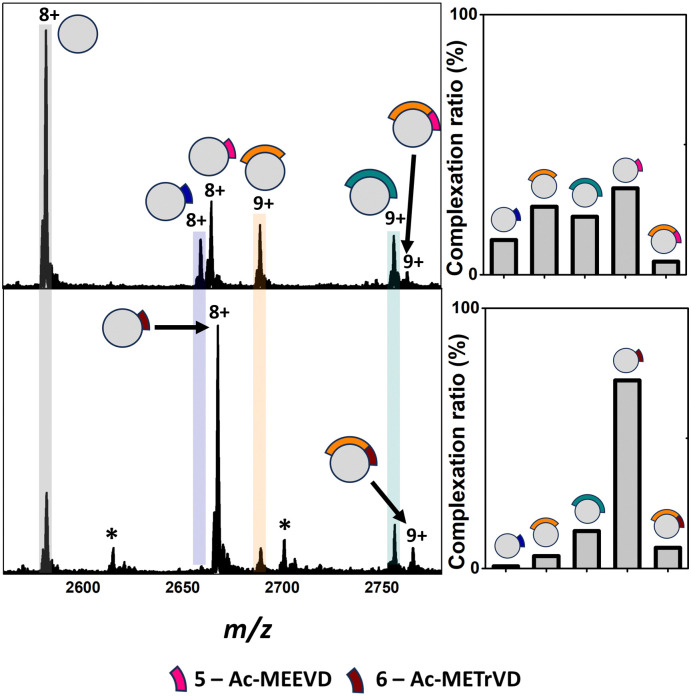
Top: Native mass spectra of TPR2A co-incubated with HSP90-C terminal peptides derived from an 18 h tryptic digestion and peptide 5 (top) or 6 (bottom). New peaks corresponding to complexes between TPR2A and peptides 5 (*m*/*z* 2663.1, [M·5 + 8H]^8+^) and 6 (*m*/*z* 2666.1, [M·6 + 8H]^8+^) can be observed alongside ternary complexes between TPR2A with both peptide 2 and 6 (*m*/*z* 2761.2, [M·2·5 + 9H]^9+^) or 2 and 6 (*m*/*z* 2763.9, [M·2·6 + 9H]^9+^). Bar graphs alongside spectra highlight the alteration of complexation ratios between TPR2A and peptides 1–3 in the presence of peptides 5 and 6, respectively. Peptide 5 had a moderate effect on the abundances of the TPR2A–1 and TPR2A–3 complexes and no impact on the TPR2A–2 complex. The presence of peptide 6 significantly reduced the abundance of TPR2A complexes with peptides 1, 2 and 3. Bottom: Peptide sequence key. *Unidentified contaminants. Full spectra can be found in Fig. S6 (ESI†).

To further demonstrate our proof of concept, we progressed to a second transient PPI between the mycobacterial chaperone, DnaK and its nucleotide exchange factor, GrpE. DnaK and GrpE are individually essential for protein homeostasis in both *M. smegmatis* (Msm) and *M. tuberculosis* (Mtb), and as such, the PPI is a promising non-canonical target for Mtb drug discovery.^21^ At the time of our DnaK-GrpE experiments, the Mtb DnaK-GrpE PPI was not defined. However, a very recent report by Xiao *et al.* disclosed a cryo-EM structure of the DnaK – GrpE PPI, where they found that the association between Mtb DnaK and two GrpE subunits occurred at three major interfacial contact regions (Fig. S7, ESI†).^22^ Having identified the three major contact regions and based on structural information, point mutations were systematically introduced to both DnaK and GrpE (Fig. S3B, ESI†). Generally, mutations on either GrpE or DnaK resulted in only minor detrimental effects on PPI formation; with the exception of DnaK mutations in contract region 1 (E236A, Y257A). This alteration in the DnaK nucleotide binding domain (NDB) not only abolished PPI formation but also the ability of DnaK to reactivate denatured luciferase. These findings highlight E236 and Y257 in DnaK as key hot-spot residues required for transient PPI formation.^22^ Our MS analysis of GrpE in isolation under native conditions revealed a charge state distribution consistent with a 25.3 kDa monomer with no dimer observed (Fig. S9, ESI†). However, when incubated with DnaK, we observed GrpE dimerisation and GrpE-DnaK PPI formation, including a 2 : 1 ternary complex, in line with the reported cryo-EM structure. This data suggested that dimerisation may only occur upon transient PPI formation with DnaK and that our experiments would be limited to interactions with a GrpE monomer. Incubation of an aliquot of DnaK-derived peptides following an 18-hour tryptic digest resulted in the binding of two DnaK peptides (7 and 8, Fig. 3 and Fig. S10 and Table S1, ESI†). Both peptides shared substantial sequence overlap and originated from the DnaK NBD and contained the key E236 hot-spot residue. LC-MS analysis of the DnaK digest revealed that overlapping peptides covering the majority of the DnaK sequence were present in the digest, including all three PPI contact regions (Fig. S3B, ESI†).

**Fig. 3:**
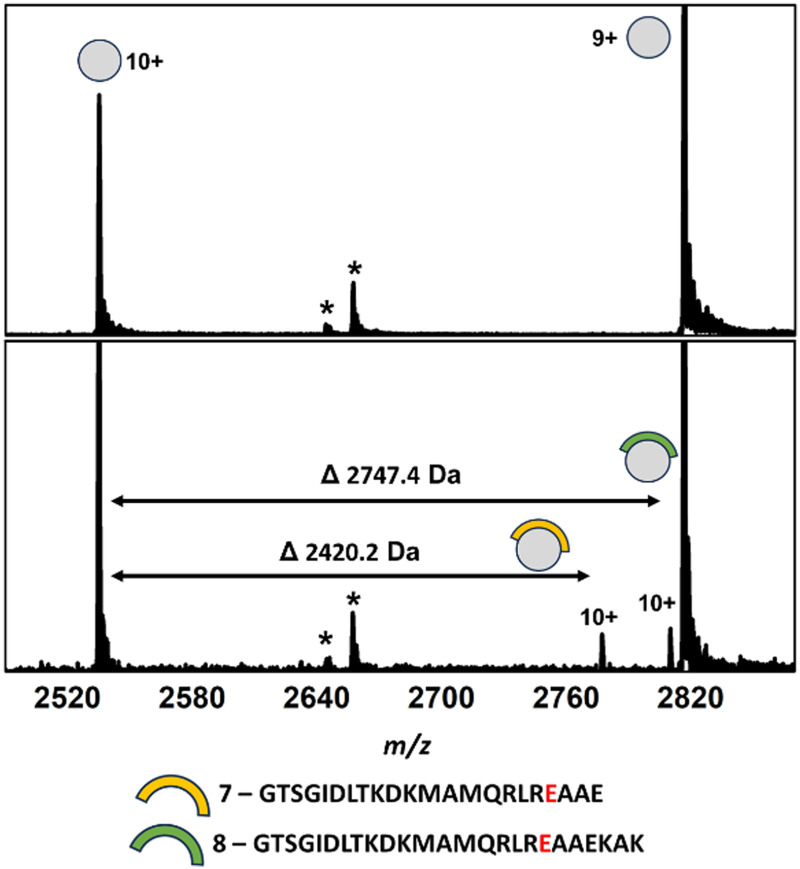
Native mass spectra of apo GrpE (top) and GrpE co-incubated with a crude mixture of DnaK peptides derived from an 18 h tryptic digestion (bottom). Complexes between GrpE and peptides 7 (*m*/*z* 2775.6, [M·7 + 10H]^10+^) and 8 (*m*/*z* 2808.3, [M·8 + 10H]^10+^) can be observed alongside apo GrpE (*m*/*z* 2533.6, [M + 10H]^10+^); 2814.9, [M + 9H]^9+^. *Unidentified contaminants. Full spectra can be found in Fig. S11 (ESI†).

Significantly, no GrpE binding was observed for any of these peptides with the exception of peptides containing E236 (Fig. S8, ESI†), supporting the notion that contact region 3 represents the key PPI interfacial region. While no single peptide containing both the E236 and Y257 residues was present, two peptides encompassing Y257 were identified, but whose GrpE binding was not observed. However, analysis of the cryo-EM structure revealed that Y257 interacts at the GrpE dimer interface, forming electrostatic interactions with residues from each GrpE subunit. Accordingly, the DnaK-dependent dimerisation of GrpE in our native MS experiment likely hampered binding of Y257-containing peptides.

In conclusion, we have demonstrated a resource-efficient approach, that, with simple sample manipulation, could qualitatively identify peptides associated with the TPR2A–HSP90-C PPI including a novel secondary interfacial region of the TPR2A–HSP90-C PPI whose interaction with TPR2A was previously unresolved through cryo-EM. Furthermore, we observed Pep-PIs representing the core DnaK-GrpE PPI region, with the subsequent cryo-EM structure providing validation for our unbiased observations. In addition to reinforcing the capacity of native MS for identifying weakly interacting interfaces, the ability to observe the secondary interfacial interaction is significant since it provides new insight into the TPR2A–HSP90 C-terminal PPI formation and disruption mechanisms. Similarly, identification of the GrpE interfacial peptides provides a starting point for the development of probes capable of modulating this biologically important PPI. Challenges associated with the generation of representative peptide libraries, alongside the technical limitations of many common biophysical techniques, significantly hinders our ability to understand and exploit these fundamental biological interactions. *In silico* methodologies have emerged perforce as the benchmark for predicting interfacial SLiMs of transient PPIs.^23^ Whilst undoubtedly powerful, the development of *in vitro* methods for elucidation and observation of interfacial interactions is critical for robust investigations. In its current form, this approach provides a qualitative assessment of interfacial epitope interactions and without peptide synthesis, is not able to derive quantitative interaction data. Users should also be cognizant of the potential for false positives and negatives resulting from the inability to control peptide concentrations. Furthermore, variability in proteolysis kinetics of different proteins can impact sequence coverage. These factors will be addressed in future studies, as we move into less well-characterised systems. However, here we have clearly demonstrated that this approach could be applied as an efficient triaging tool to inform targeted peptide synthesis, phage library construction and scanning mutagenesis experiments, for full characterisation of transient PPI interfaces. Similarly, this approach can act as a powerful orthogonal method to improve the efficiency of other common structural methodologies to provide structural insight into the plethora of biologically relevant transient PPIs and support structure-based drug design of PPI modulators.

CGLV, ALE and DJC designed the study, CGLV conducted MS experiments. AC and RM expressed and purified proteins, FA and BGdlT produced synthetic peptides.

Authors acknowledge support from the BBSRC (Grant No. BB/R013993/1), UCT, Rhodes University, the South African Research Chairs Initiative of the DSI and NRF (Grant No. 98566), the NRF CPRR (Grant No 129262), and Future Leaders – African Independent Research (FLAIR), a partnership between the AAS and the Royal Society that is funded by the UK Government as part of the Global Challenge Research Fund (GCRF).

## Conflicts of interest

There are no conflicts to declare.

## Supplementary Material

CC-060-D4CC01251H-s001
