# Supplementary material for: A native mass spectrometry approach to qualitatively elucidate interfacial epitopes of transient protein–protein interactions
Source: Chem Commun (Camb). 2024 May 13;60(45):5844–7. doi: 10.1039/d4cc01251h (PMC11139139; doi:10.1039/d4cc01251h)
Supplement: CC-060-D4CC01251H-s001 [file CC-060-D4CC01251H-s001.pdf]

Electronic Supplementary Material (ESI) for ChemComm.
This journal is © The Royal Society of Chemistry 2024

A Native Mass Spectrometry Approach to Qualitatively Elucidate Interfacial Epitopes of Transient Protein-Protein Interactions

Experimental Procedures

1. Protein expression and purification

His-tagged TPR2A domain of human HOP and the His-tagged human HSP90αC domain were produced in the *E. coli* BL21(DE3) Codon+ strain from the pQE80L plasmid with an N-terminal hexahistidine tag. For both proteins, the transformed bacterial culture was grown in 2xYT medium [1.6 % (w/v) tryptone, 1 % (w/v) yeast extract and 0.5 % [w/v] NaCl)) to an OD_{600} of 0.6, and protein production was induced with 0.5 mM isopropyl β-d-1-thiogalactopyranoside (IPTG) for 18 hours at 25°C. Cells were harvested at 6000x*g* for 15 minutes at 4°C, and the cell pellets were resuspended in His lysis buffer [50 mM NaH_2PO_4 pH 7.5, 300 mM NaCl, with 1 mg/ml lysozyme, 0.1 % (w/v) Triton-X and 1 mM phenylmethylsulphonyl fluoride (PMSF)]. The cell lysates were centrifuged at 16000x*g* for 30 mins at 4°C, and the supernatants were loaded onto Ni^{+2}-charged HisPur™ Ni-NTA Resin (Thermo Fisher Scientific, Cat # 88222), followed by overnight incubation at 4°C on a rocker. Washing was performed with cold wash buffer (50 mM NaH_2PO_4 pH 7.5 300 mM NaCl) containing 2-5 mM imidazole, and proteins were eluted with cold elution buffer (50 mM NaH_2PO_4 pH7.5, 300 mM NaCl, 200 mM imidazole). Eluted proteins were buffer exchanged into storage buffer (25 mM NaH_2PO_4 pH 7.5, 150 mM NaCl, 150 mM KCl) using a 3 kDa MWCO filter prior to downstream analysis. *Mycobacterium tuberculosis* (Mtb) DnaK and GrpE proteins were produced in *E. coli* BL21 (DE3) Codon+ strain as fusion proteins with an N-terminal His-SUMO tag from the pCA258 backbone (a kind gift of Matthias Mayer, ZMBH, Heidelberg). His-SUMO-DnaK and His-SUMO-GrpE proteins were purified by Ni-NTA chromatography as described above and buffer exchanged into storage buffer (25 mM NaH_2PO_4 pH 7.5, 150 mM NaCl, 150 mM KCl). The His-SUMO tag was removed by addition of His-tagged SUMO protease (purified in house) for 4 hours at 4 °C with gentle rotation. Protein mixtures were subjected to a second round of Ni-NTA chromatography to remove the SUMO protease and His-SUMO tag, and then passed through a 10 kDa MWCO filter to obtain untagged DnaK and GrpE proteins.

2. Sample Preparation

a. Protein desalting

TPR2A, HSP90-C, DnaK and GrpE samples were subjected to two rounds of buffer exchange into 100 mM NH_4OAc using Zeba Spin Desalting Column (Thermo Fisher Scientific). Concentration of all proteins ere adjusted to stock solutions of 250 µM.

b. HSP90-C digestion

100 µL of agarose supported trypsin (Thermo Fischer) was washed three times with 500 µL NH_4OAc buffer (100 mM), and finally resuspended in 200 µL of the same NH_4OAc buffer. 50 µL of the desalted HSP90-C or DnaK stock solutions (250 µM) were added to this trypsin suspension. The mixture was incubated at 37 °C on a shaking heat block. Digest aliquots were removed at indicated intervals and used directly for native MS analysis.

c. Preparation of Peptide 10 and 11 stocks

Stock solutions (250 µM) of peptides **5** and **6** were prepared in 100 mM NH_4OAc.

d. Sample preparation for Native MS

TPR2A-HSP90C: 1 µL of the TPR2A stock (250 µM) was utilised for analysis and made up to a final volume of 20 µL (final protein concentration 12.5 µM). HSP90-C peptide binding experiments were prepared by mixing 2 µL aliquots of the digest mixtures with 1 µL of the TPR2A stock, and 17 µL of 100 mM NH_4OAc. Similarly, binding competition samples were prepared HSP90-C peptide binding experiments were prepared by mixing 2 µL aliquots of the digest mixtures with 1 µL of the peptide stock solution (final conc. 12.5 µM) 1 µL of the TPR2A stock, and 16 µL of 100 mM NH_4OAc.

GrpE-DnaK: GrpE – DnaK PPI formation experiments were conducted by mixing 2 µL of the GrpE stock (250 µM) with 1 µL of the DnaK stock (250 µM) and made up to a final volume of 20 µL in 100 mM NH_4OAc (final protein concentration 25 µM GrpE and 12.5 µM DnaK). GrpE DnaK peptide binding experiments were prepared by mixing 4 µL aliquots of the digest mixtures with 2 µL of the GrpE stock, and 14 µL of 100 mM NH_4OAc.
All samples were made up in 96-well plates and held at 4 °C prior to MS analysis.

3. Mass Spectrometry

Native MS were obtained on a 12T SolariX 2XR or 7T FTICR (Bruker Daltonics). Ionisation was achieved using a NanoMate nESI infusion robot (TriVersa Biosciences), sampling from a 96-well plate. Typically, a nanoelectrospray voltage of 1.55 kV was used and backing pressure was adjusted to maintain stable electrospray. Instrument conditions were optimised for transmission of native protein ions. Typical source optics were: capillary exit 250V, deflector plate 230V, funnel 1 210V, skimmer 1 50V, funnel2 15 and skimmer 2 8V. Native mass spectra were acquired as the sum of 200 1 MegaWord FID transients. DataAnalysis software (Bruker Daltonics) was used for native MS analysis.

LC-MS data was acquired on a Synapt G2 q-TOF mass spectrometer, coupled to an Acquity UPLC (Waters) equipped with a 50 x 2.1 mm C18 reverse phase column (Phenomenex). Chromatography was performed at 298K. Mobile phase A (0.1% TFA in H_2O) and mobile phase B (0.1% TFA in CH_3CN) were set at 95% (A) and 5% (B) with a gradient running to 5% (A) and 95% (B) over 20 minutes. LC-MS data were processed using MassLynx v4.0 (Waters).

4. Supplementary Figures

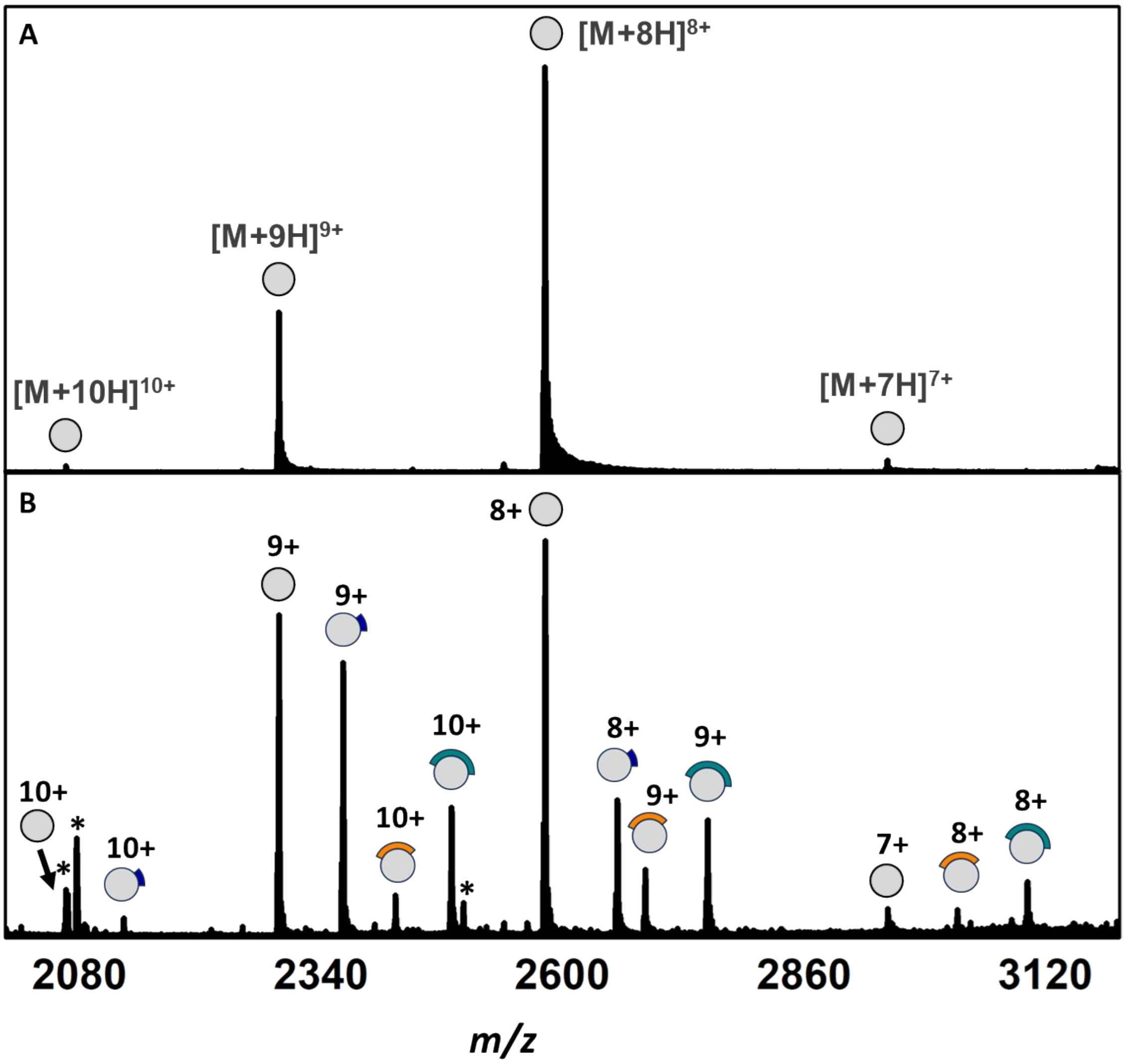

Figure S1. A: Charge state distribution of apo TPR2A in the native state (*m/z* 2064.4, $[M+10H]^{10+}$; *m/z* 2293.6, $[M+9H]^{9+}$; *m/z* 2580.2, $[M+8H]^{8+}$; *m/z* 2948.7, $[M+7H]^{7+}$ **B:** Full native MS spectrum of TPR2A, incubated with HSP90-C terminal peptides derived from 18h of tryptic digestion. No binding was observed for species in the 7+ charge state. *Unidentified low charge state peptide contaminants, including overlap with the 10+ apo TPR2A peak in spectrum B.

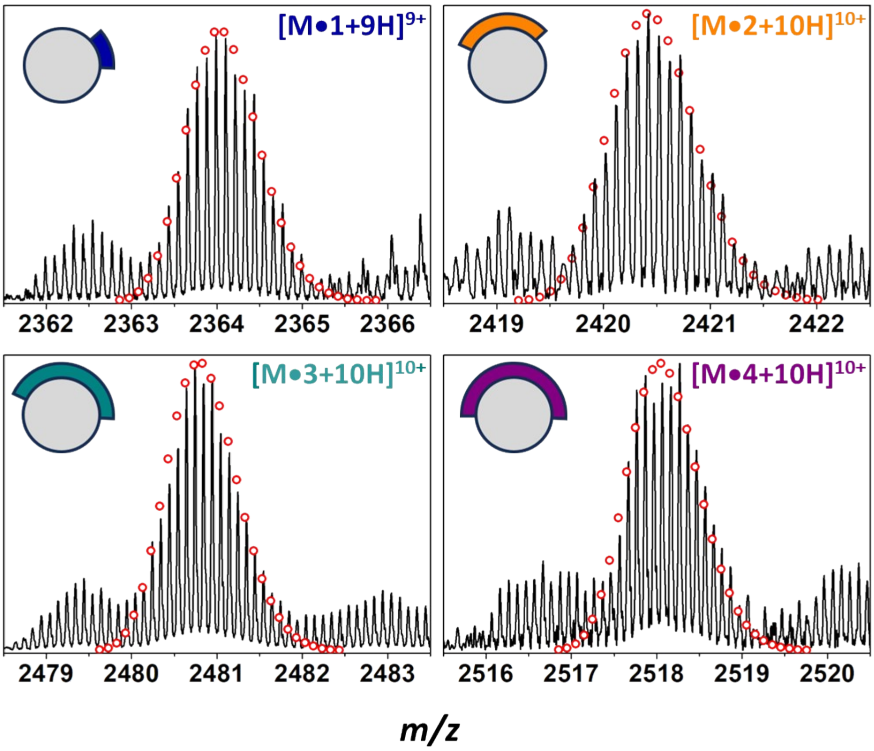

Fig. S2. High-resolution ESI FT-ICR MS analysis of the isotope distributions of TPR2A in complex with peptides **1 – 4**. For each species, the calculated theoretical isotope distribution for the TPR2A-peptide complex is overlaid as a scatterplot and are representative of the following molecular formulae: 1. $[C_{937}H_{1474}N_{264}O_{288}S_7 + 9H]^{9+}$; 2. $[C_{1057}H_{1662}N_{296}O_{341}S_7 + 10H]^{10+}$; 3. $[C_{1081}H_{1699}N_{301}O_{352}S_8 + 10H]1^{10+}$; 4. $[C_{1098}H_{1731}N_{305}O_{355}S_9 + 10H]^{10+}$.These data facilitated unambiguous Δ *m/z* assignment and binding peptide identification.

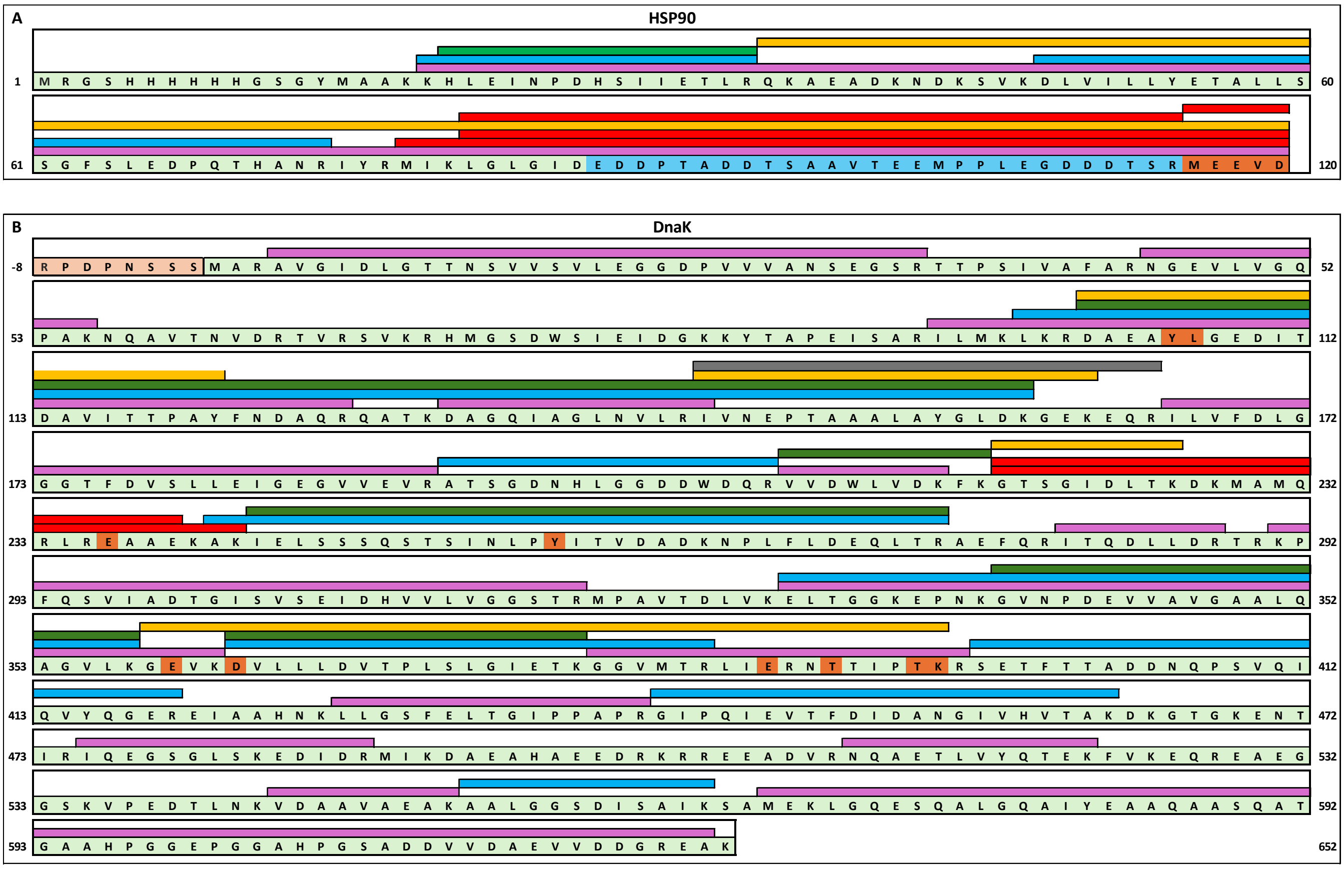

Fig. S3. Depiction of the sequence overage of peptides derived from tryptic digestion of HSP90-C (**A**) and DnaK (**B**) respectively. Peptides coloured red indicate binding to either TPR2A (**A**) or GrpE (**B.**). Colours used for non-binding peptides are varied, only for the purposes of figure clarity. **A**) Residues highlighted in orange, indicate known interfacial associating epitope (MEEVD). Residues highlighted in blue, correlate with previously unreported TPR2A interacting region.[1] **B**). Residues highlighted in orange, correlate to those mutated by Li and co-workers to assess influence of various contact areas of the DnaK-GrpE PPI.[2]

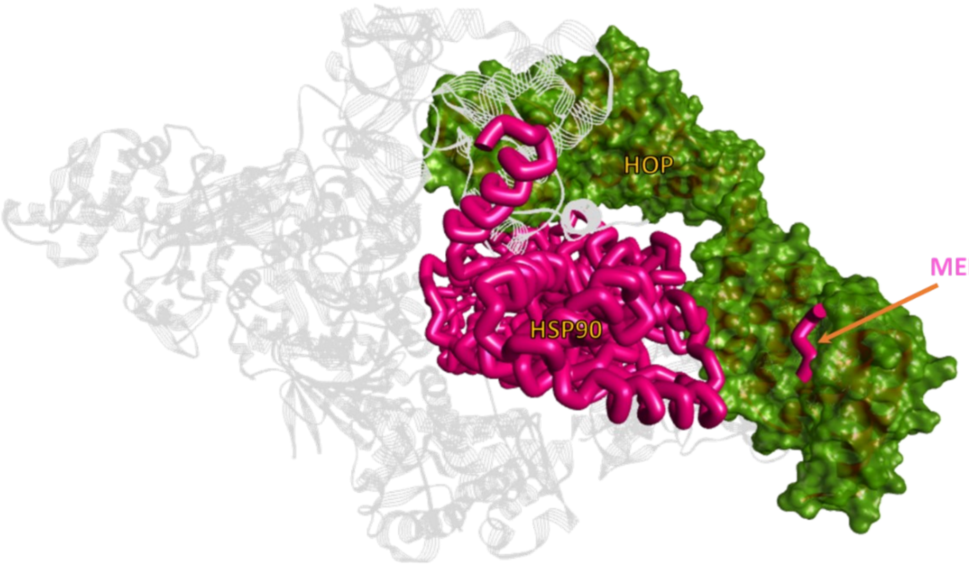

Fig. S4. Cryo-EM structure of the GR–HSP90–HSP70–HOP complex (PDB 7KW7).[1] The HSP90 (pink tube) association with HOP(green van der Waals surface), is highlighted, while the HSP70 and GR are greyed out for clarity. In this structure, the primary interfacial interaction between TPR2A and MEEVD has been resolved. However, the sequence, which connects MEEVD to the reminder of HSP90 is not included in the structure. This omitted region corresponds closely to **peptide 2**, identified in this study as a TPR2A binder.

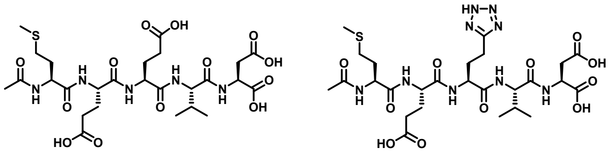

5. Ac-MEEVD-OH

6. Ac-METrVD-OH

Figure S5. Competitively binding peptides **5** and **6**

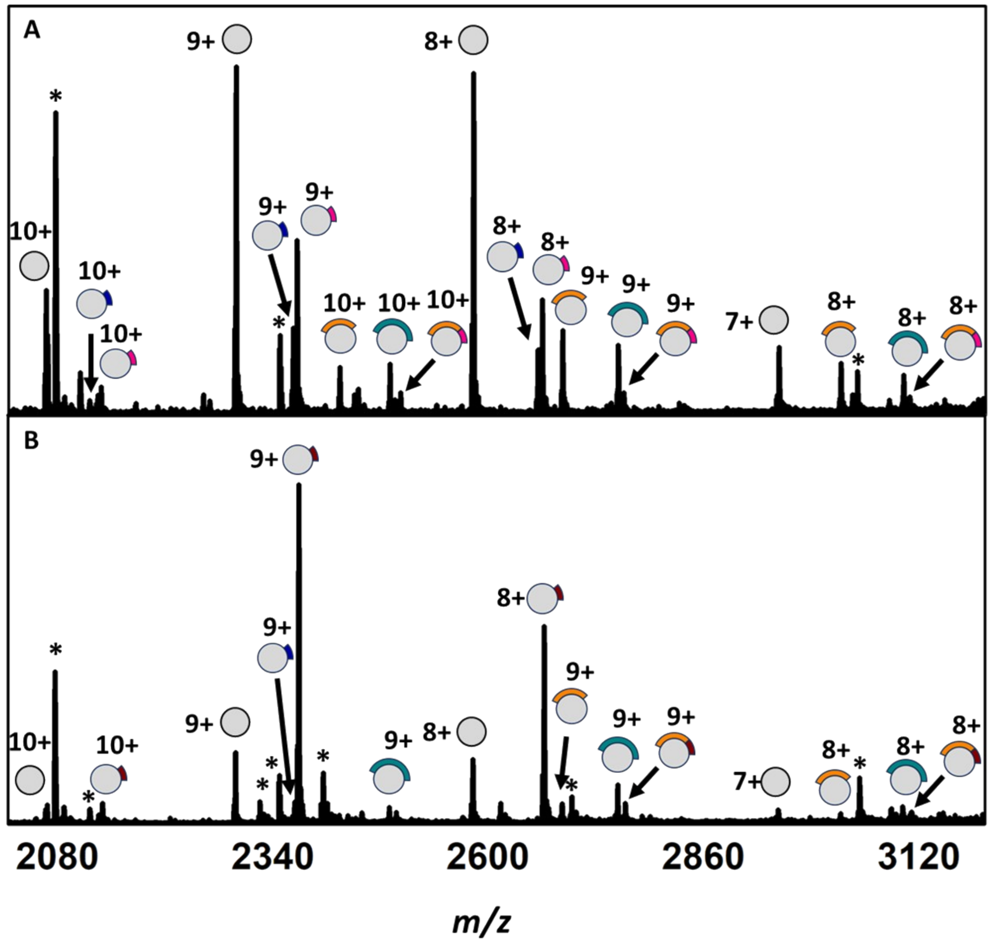

Figure S6. Competitively binding peptides **5** and **6.** Charge state distribution of apo TPR2A in the native state (*m/z* 2064.4, $[M+10H]^{10+}$; *m/z* 2293.6, $[M+9H]^{9+}$; *m/z* 2580.2, $[M+8H]^{8+}$; *m/z* 2948.7, $[M+7H]^{7+}$ **B:** Full native MS spectrum of TPR2A, incubated with HSP90-C terminal peptides derived from 18h of tryptic digestion. No binding was observed for species in the 7+ charge state. *Unidentified low charge state peptide contaminants, including overlap with the 10+ apo TPR2A peak in spectrum B.

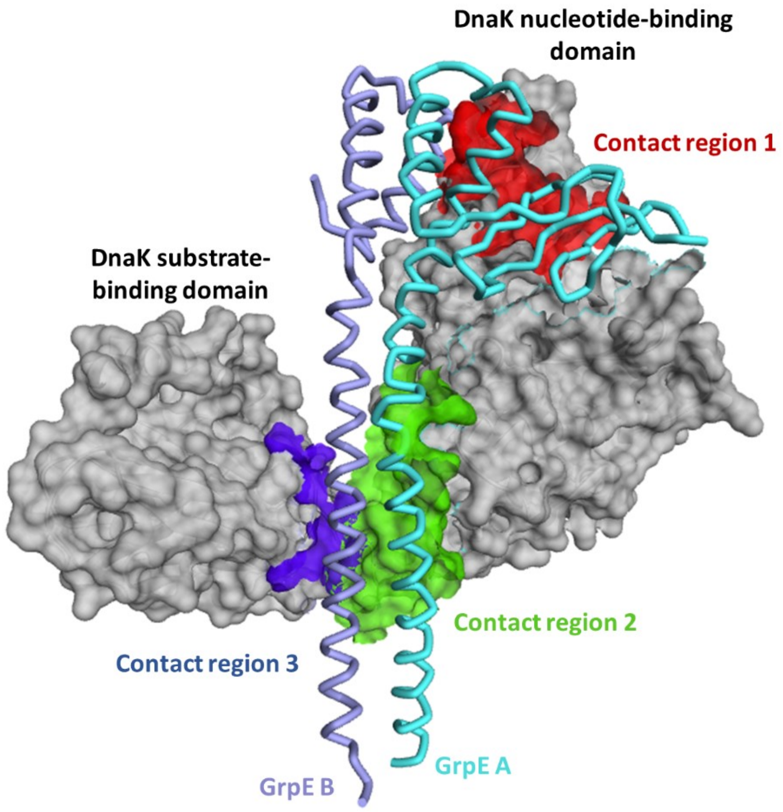

Fig. S7. Cryo-EM structure of DnaK (grey) bound to two subunits of GrpE (blue and purple, PDB 8GB3).[2] This PPI is formed through three major contact regions as shown. Mutational analysis showed that a dual E236A and Y257A mutation abolished PPI formation and DnaK chaperone activity.

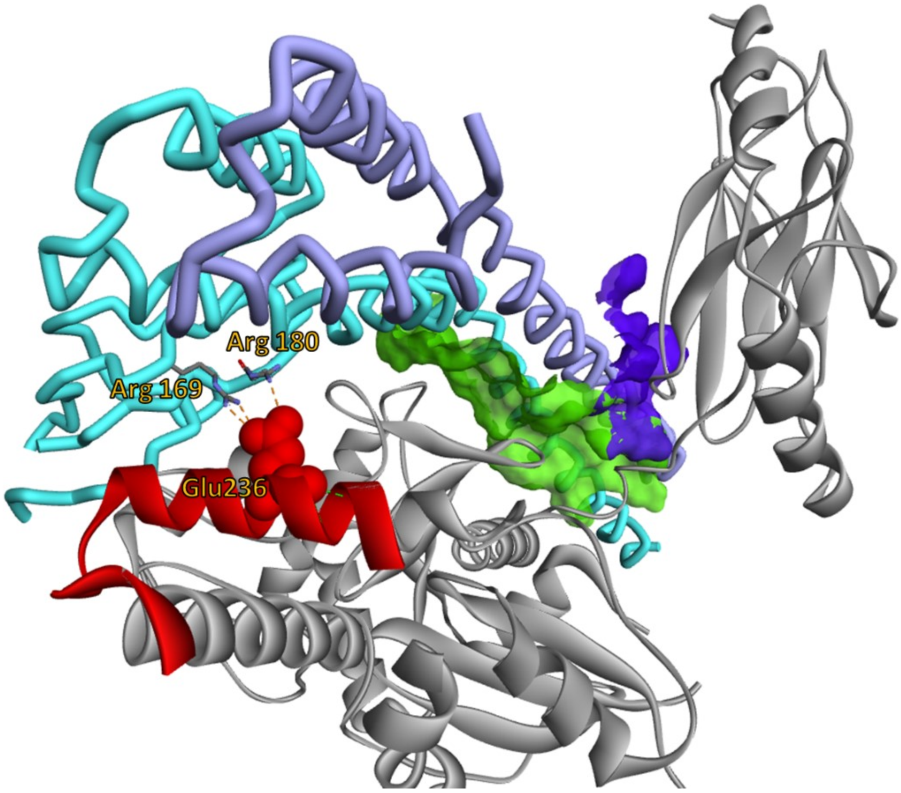

Fig. S8. Zoomed in portion of contact region 1. Overlapping peptides **7** and **8** were found in the alpha helical region highlighted in red, and includes key E236 residue, which forms salt bridges with Arg169 and 180 on the same GrpE monomer (blue)

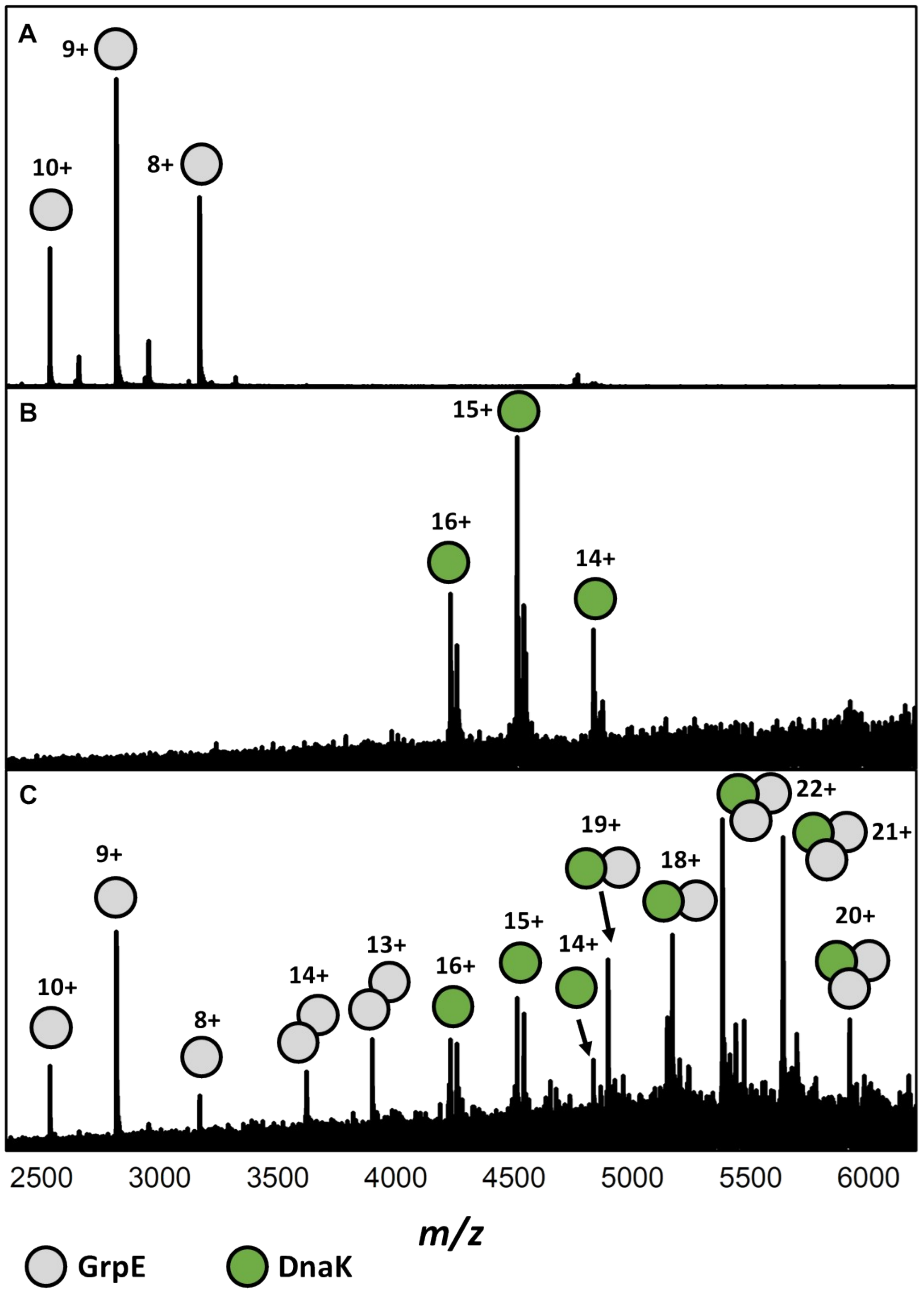

Figure S9. A: Charge state distribution of apo GrpE in the native state (*m/z* 2533.6, $[M+10H]^{10+}$; *m/z* 2814.9, $[M+9H]^{9+}$; *m/z* 3166.7 $[M+8H]^{8+}$), showing the formation of a monomeric species under our native MS conditions. **B:** Charge state distribution of apo DnaK in the native state (*m/z* 4227.9, $[M+16H]^{16+}$; *m/z* 4509.7 $[M+15H]^{15+}$; *m/z* 4831.8 $[M+14H]^{14+}$). **C**: Combining GrpE and DnaK in a 2:1 molar ratio resulted in the formation of a 1:1 GrpE-DnaK complex (*m/z* 4892.5, $[M+19H]^{19+}$; *m/z* 5164.3 $[M+18H]^{18+}$) and a 2:1 ternary complex as per the reported cryo-Em structure (*m/z* 5375.9, $[M+22H]^{22+}$; *m/z* 5631.8 $[M+21H]^{21+}$; *m/z* 5913.4 $[M+20H]^{20+}$). Furthermore, under these conditions we observed dimeric GrpE (*m/z* 3617.8, $[M+14H]^{14+}$; *m/z* 3895.9 $[M+13H]^{13+}$).

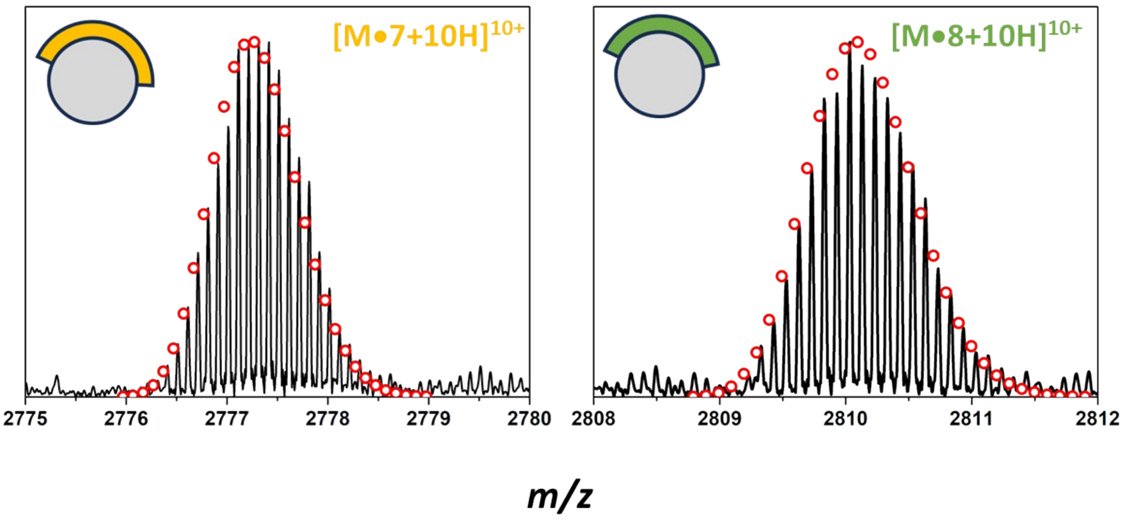

Fig. S10. High-resolution ESI FT-ICR MS analysis of the isotope distributions of GrpE in complex with peptides **7** and **8**. For each species, the calculated theoretical isotope distribution for the GrpE-peptide complex is overlaid as a scatterplot and are representative of the following molecular formulae: 1. $[C_{1165}H_{18174}N_{355}O_{423}S_4 + 10H]^{10+}$; 2. $[C_{1210}H_{1916}N_{2360}O_{426}S_4 +10H]^{10+}$. These data facilitated unambiguous Δ *m/z* assignment and binding peptide identification.

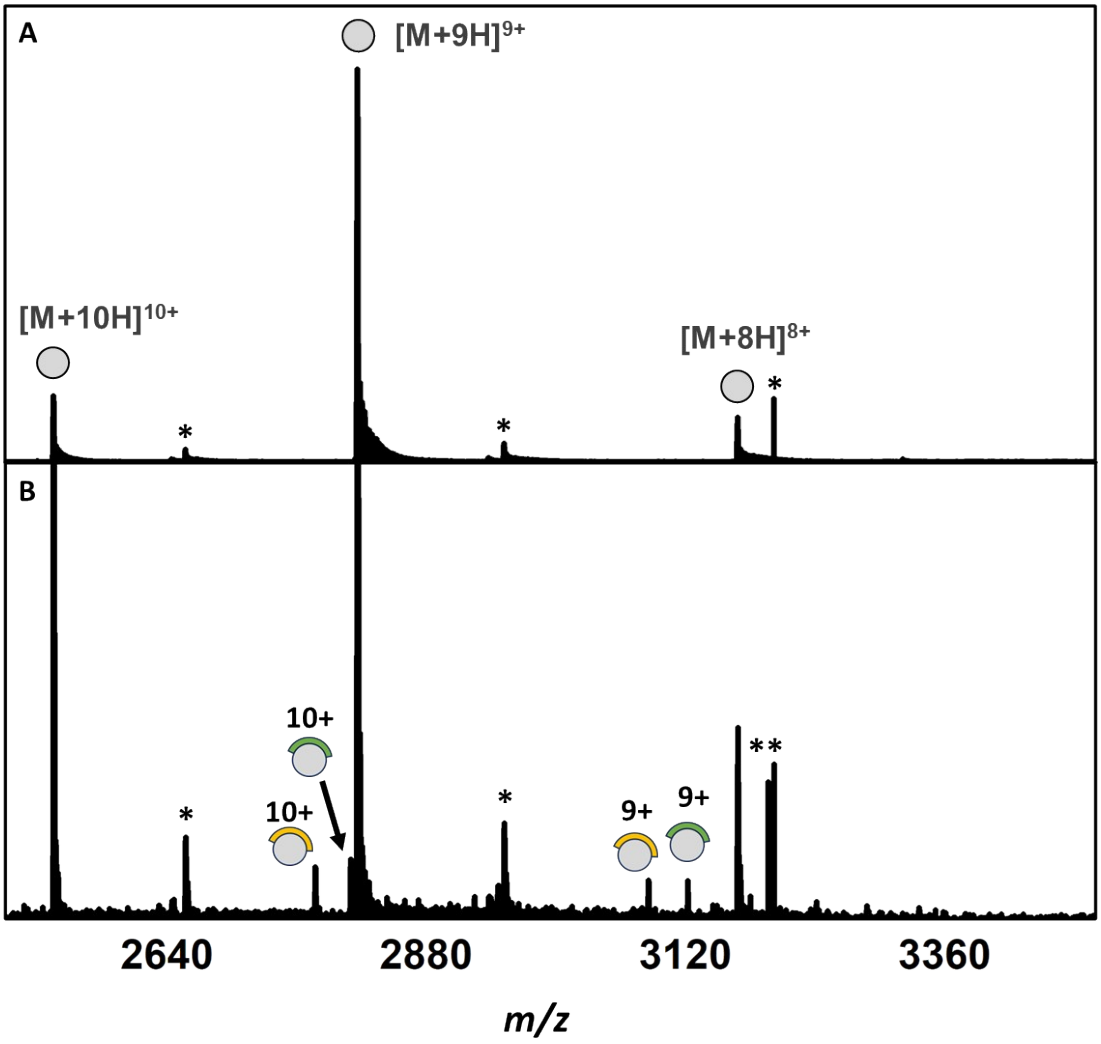

Figure S11 A: Charge state distribution of apo GrpE in the native state (*m/z* 2533.6, $[M+10H]^{10+}$; *m/z* 2814.9, $[M+9H]^{9+}$; *m/z* 3166.7 $[M+8H]^{8+}$. **B** Full native MS spectrum of GrpE, incubated with DnaK peptides derived from 18h of tryptic digestion. No binding was observed for species in the 8+ charge state.*Unidentified contaminants.

5. Data Analysis

For each spectrum a mass list and accompanying peak areas was generated using the FTMS algorithm (S/N threshold of 5). Protein – Peptide complexes were identified via Δ*m/z* as compared to apo protein at each charge state (**Table S1**). The area of each peak corresponding to a TPR2A-peptide complex from each of the three native charge states (10+, 9+ and 8+) was combined. TPR2A-peptide association was calculated from the summed peak area of each species as a percentage of the total peak area for each species. TPR2A-peptide associations for each individual charge state was calculated from the individual peak area as a percentage of the total peak area at that charge state (**Table S2**). Figures are the average of two replicates.

A list of possible tryptic cleavage products was generated using Expasy PeptideMass[3] with missed cleavages set to 5. The crude mixture following 18 hours of tryptic digestion was subjected to UPLC-MS, where tryptic cleavage product were identified by their mass signature (**Figure S3**).

6. Supplementary Tables and Figures

Table S1. Δ*m/z* of TPR2A and GrpE binding peptides

			Monoisotopic mass	Δ*m/z* 10+	9+	8+
HSP90	**Peptide 1**	**MEEVD**	621.23	**62.123**	**69.03**	**77.65**
	**Peptide 2**	**LGLGIDEDDPTADDTSAAVTEEMPPLEGDDDTSR**	3546.53	**354.653**	**394.06**	**443.32**
	**Peptide 3**	**LGLGIDEDDPTADDTSAAVTEEMPPLEGDDDTSRMEEVD**	4149.75	**414.98**	**461.08**	**518.72**
	**Peptide 4**	**MIKLGLGIDEDDPTADDTSAAVTEEMPPLEGDDDTSRMEEVD**	4521.97	**452.20**	**502.44**	**565.25**
	**Peptide 5**	**Ac-MEEVD-OH**	663.24	**66.32**	**73.69**	**82.91**
	**Peptide 6**	**Ac-METrVD-OH**	687.26	**68.73**	**76.36**	**85.91**
DnaK	**Peptide 7**	**GTSGIDLTKDKMAMQRLREAAE**	2420.2	**242.0**	**268.9**	
	**Peptide 8**	**GTSGIDLTKDKMAMQRLREAAEKAK**	2747.4	**274.7**	**305.3**	

Table S1 - Peak areas of each TPR2A - peptide complex at 10+ 9+ and 8+ charge states

TPR2A and 1 hour trypsin digest

Associting Peptide	Peak Area 10+	Peak Area 9+	Peak Area 8+	Sum for each complex	Complexation ratio over individual charge states (%) 10+	9+	8+	Complexation ratio over all charge states (%)
Peptide 1	0	1053439	910818	**1964257**	**0**	**4.44**	**6.08**	**4.04**
Peptide 2	188027	628819	0	**816846**	**1.91**	**2.65**	**0**	**1.68**
Peptide 3	8164246	20644026	12853107	**41661379**	**83.1**	**86.9**	**85.8**	**85.8**
Peptide 4	1476830	1406567	1219093	**4102490**	**15.0**	**5.92**	**8.14**	**8.45**
**Sum for each charge state**	**9829103**	**23732851**	**14983018**					
	**Total**		**48544972**					

TPR2A and 2 hour trypsin digest

Associating Peptide	Peak Area 10+	Peak Area 9+	Peak Area 8+	Sum for each complex	Complexation ratio over individual charge states (%) 10+	9+	8+	Complexation ratio over all charge states (%)
Peptide 1	215006	2599536	4124846	**6939388**	**1.06**	**5.59**	**11.7**	**6.81**
Peptide 2	849615	3284724	2160752	**6295091**	**4.19**	**7.07**	**6.12**	**6.17**
Peptide 3	18174782	39100112	27607906	**84882800**	**89.7**	**84.2**	**78.3**	**83.2**
Peptide 4	1024690	1442473	1376475	**3843638**	**5.06**	**3.11**	**3.90**	**3.77**
**Sum for each charge state**	**20264093**	**46426845**	**35269979**					
		**Total**	**101960917**					

TPR2A and 18 hour trypsin digest

Associating Peptide	Peak Area 10+	Peak Area 9+	Peak Area 8+	Sum for each complex	Complexation ratio over individual charge states (%) 10+	9+	8+	Complexation ratio over all charge states (%)
Peptide 1	284422	3199550	2600608	**6084580**	**13.6**	**33.4**	**52.8**	**36.6**
Peptide 2	640074	1980404	1131010	**3751488**	**30.7**	**20.7**	**22.9**	**22.6**
Peptide 3	1163291	4407053	1198269	**6768613**	**55.7**	**45.9**	**24.3**	**40.8**
Peptide 4	0	0	0	**0**	**0**	**0**	**0**	**0**
**Sum for each charge state**	**2087787**	**9587007**	**4929887**					
		**Total**	**16604681**					

TPR2A and 18 hour trypsin digest + Ac-MEEVD-OH

Associating Peptide	Peak Area 10+	Peak Area 9+	Peak Area 8+	Sum for each complex	Complexation ratio over individual charge states (%) 10+	9+	8+	Complexation ratio over all charge states (%)
Peptide 1	290112	903797	2360950	**3554859**	**6.98**	**6.97**	**24.5**	**13.3**
Peptide 2	1505832	3548110	1939216	**6993158**	**36.3**	**27.4**	**20.2**	**26.1**
Peptide 3	1634420	2858139	1455786	**5948345**	**39.4**	**22.1**	**15.2**	**22.3**
Ac-MEEVD-OH	445425	4853345	3570962	**8869732**	**10.7**	**37.4**	**37.2**	**33.2**
Ac-MEEVD-OH + Peptide 2	275890	797004	275890	**1348784**	**6.64**	**6.15**	**2.87**	**5.05**
**Sum for each charge state**	**4151679**	**12960395**	**9602804**					
		**Total**	**26714878**					

TPR2A and 18 hour trypsin digest + Ac-METrVD-OH

Associating Peptide	Peak Area 10+	Peak Area 9+	Peak Area 8+	Sum for each complex	Complexation ratio over individual charge states (%) 10+	9+	8+	Complexation ratio over all charge states (%)
Peptide 1	0	219919	0	**219919**	**0**	**1.53**	**0**	**0.847**
Peptide 2	0	736447	481048	**1217495**	**0**	**5.14**	**4.81**	**4.69**
Peptide 3	705668	2207969	804376	**3718013**	**43.1**	**15.4**	**8.05**	**14.3**
Ac-METrVD-OH	573528	10035904	8152358	**18761790**	**35.0**	**69.9**	**81.6**	**72.2**
Ac-METrVD-OH + Peptide 2	357600	1138675	558534	**2054809**	**21.8**	**7.94**	**5.59**	**7.91**
**Sum for each charge state**	**1636796**	**14338914**	**9996316**					
		**Total**	**25972026**					

References

1 R. Y. R. Wang, C. M. Noddings, E. Kirschke, A. G. Myasnikov, J. L. Johnson and D. A. Agard, *Nature*, 2022, **601**, 460–464.

2 X. Xiao, A. Fay, P. Santos Molina, A. Kovach, M. S. Glickman and H. Li, *Nat. Commun.*, 2024, **15**, 660.

3 M. R. Wilkins, I. Lindskog, E. Gasteiger, A. Bairoch, J. Sanchez, D. F. Hochstrasser and R. D. Appel, *Electrophoresis*, 1997, **18**, 403–408.
